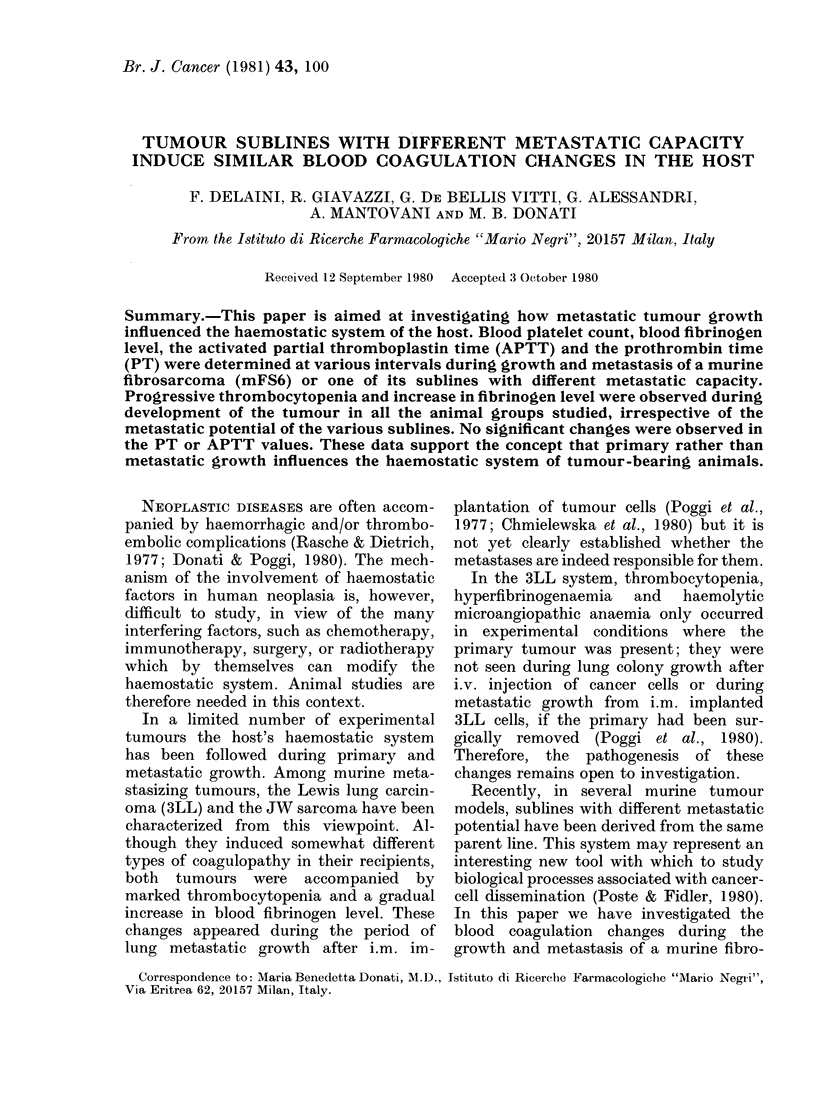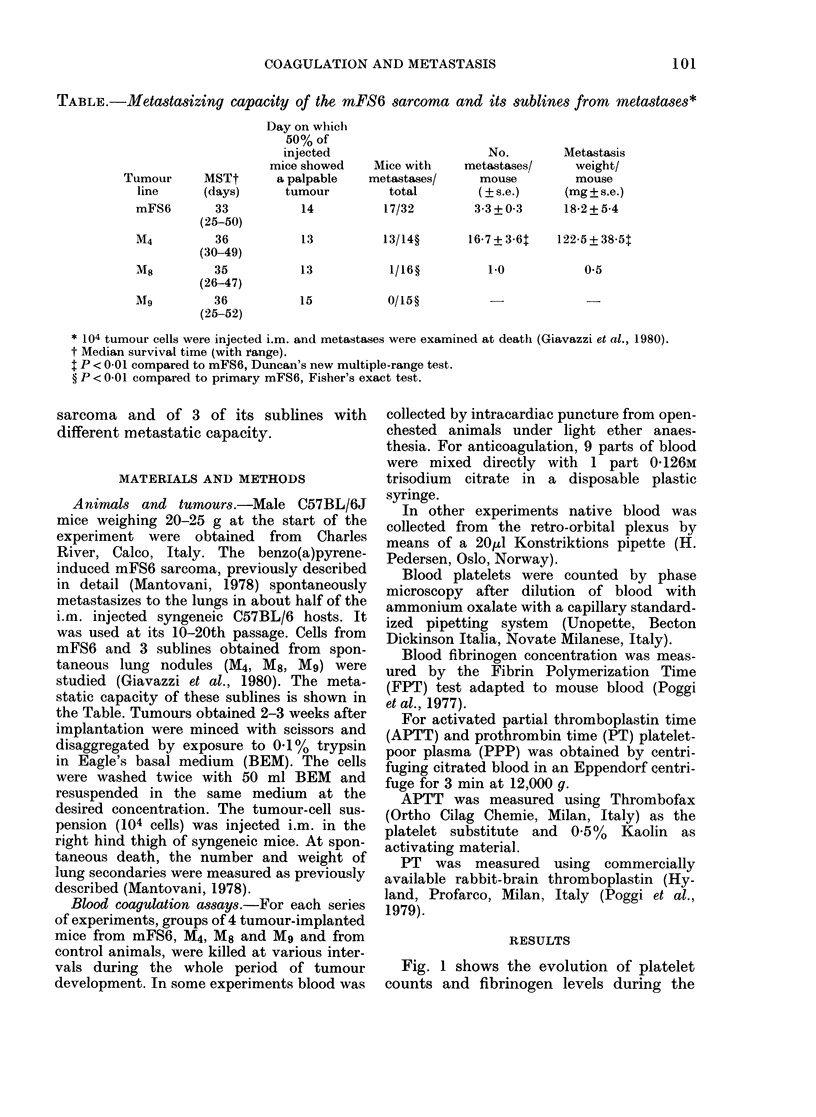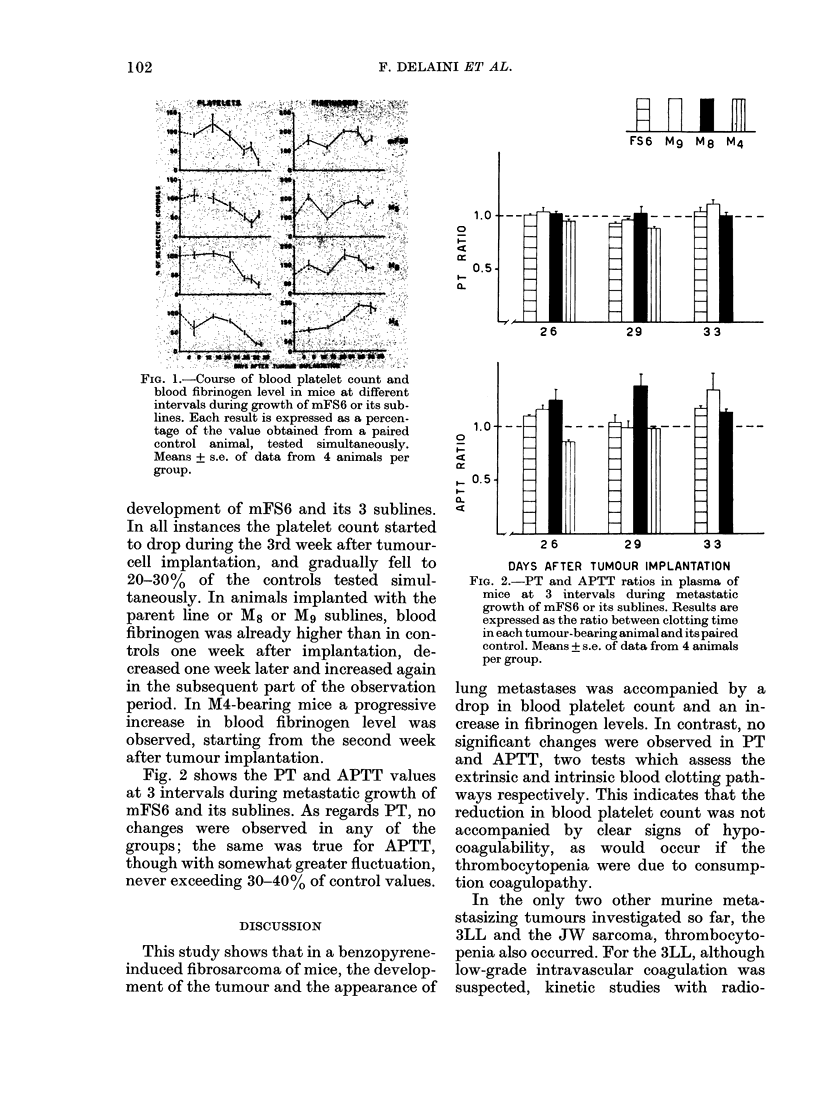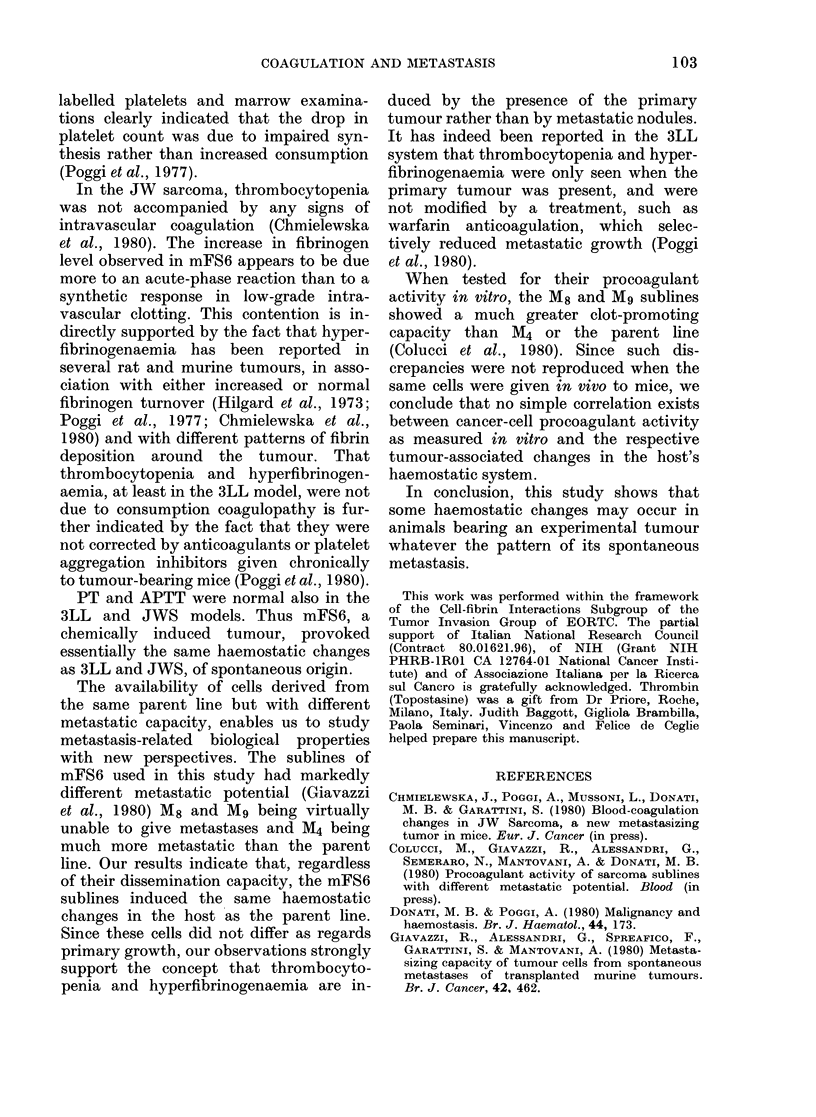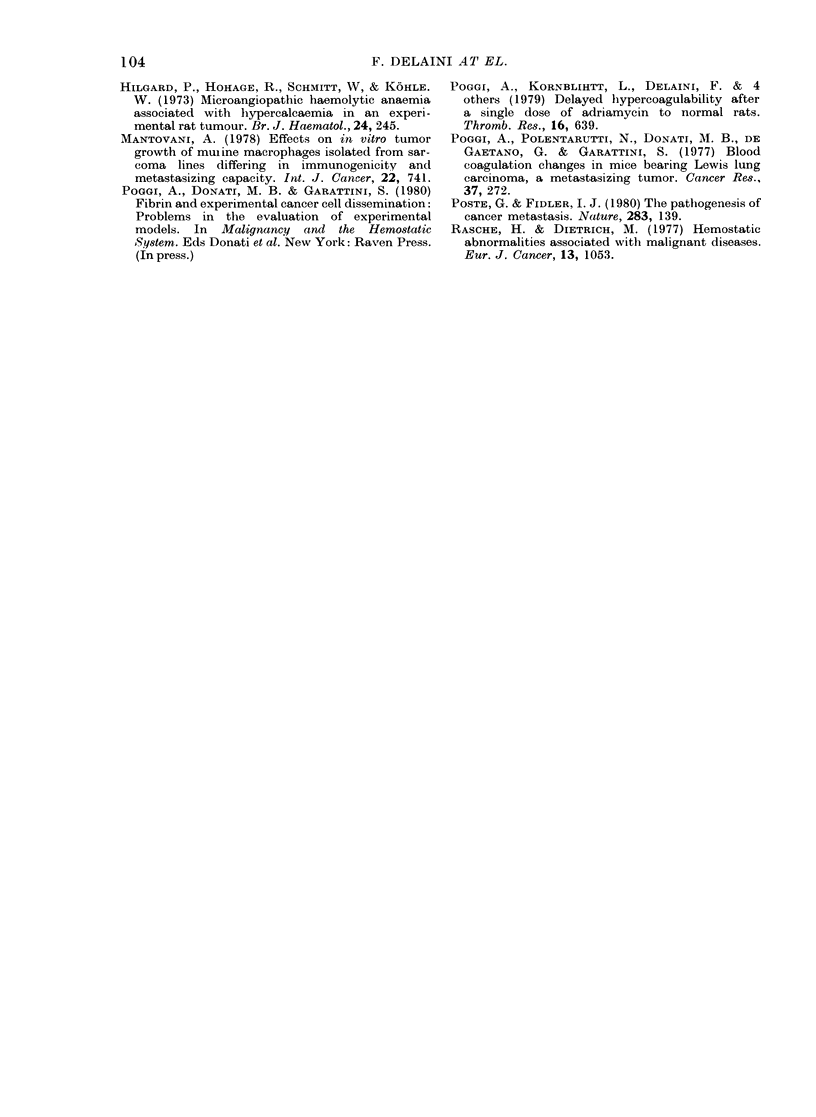# Tumour sublines with different metastatic capacity induce similar blood coagulation changes in the host.

**DOI:** 10.1038/bjc.1981.14

**Published:** 1981-01

**Authors:** F. Delaini, R. Giavazzi, G. De Bellis Vitti, G. Alessandri, A. Mantovani, M. B. Donati

## Abstract

This paper is aimed at investigating how metastatic tumour growth influenced the haemostatic system of the host. Blood platelet count, blood fibrinogen level, the activated partial thromboplastin time (APTT) and the prothrombin time (PT) were determined at various intervals during growth and metastasis of a murine fibrosarcoma (mFS6) or one of its sublines with different metastatic capacity. Progressive thrombocytopenia and increase in fibrinogen level were observed during development of the tumour in all the animal groups studied, irrespective of the metastatic potential of the various sublines. No significant changes were observed in the PT or APTT values. These data support the concept that primary rather than metastatic growth influences the haemostatic system of tumour-bearing animals.


Br. J. Cancer (1981) **43**, 100

### TUMOUR SUBLINES WITH DIFFERENT METASTATIC CAPACITY INDUCE SIMILAR BLOOD COAGULATION CHANGES IN THE HOST

F. DELAINI, R. GIAVAZZI, G. DE BELLIS VITTI, G. ALESSANDRI, A. MANTOVANI AND M. B. DONATI

*From the Istituto di Ricerche Farmacologiche "Mario Negri", 20157 Milan, Italy*

Received 12 September 1980 Accepted 3 October 1980

**Summary.—This paper is aimed at investigating how metastatic tumour growth influenced the haemostatic system of the host. Blood platelet count, blood fibrinogen level, the activated partial thromboplastin time (APTT) and the prothrombin time (PT) were determined at various intervals during growth and metastasis of a murine fibrosarcoma (mFS6) or one of its sublines with different metastatic capacity. Progressive thrombocytopenia and increase in fibrinogen level were observed during development of the tumour in all the animal groups studied, irrespective of the metastatic potential of the various sublines. No significant changes were observed in the PT or APTT values. These data support the concept that primary rather than metastatic growth influences the haemostatic system of tumour-bearing animals.**

NEOPLASTIC DISEASES are often accompanied by haemorrhagic and/or thromboembolic complications (Rasche & Dietrich, 1977; Donati & Poggi, 1980). The mechanism of the involvement of haemostatic factors in human neoplasia is, however, difficult to study, in view of the many interfering factors, such as chemotherapy, immunotherapy, surgery, or radiotherapy which by themselves can modify the haemostatic system. Animal studies are therefore needed in this context.

In a limited number of experimental tumours the host's haemostatic system has been followed during primary and metastatic growth. Among murine metastasizing tumours, the Lewis lung carcinoma (3LL) and the JW sarcoma have been characterized from this viewpoint. Although they induced somewhat different types of coagulopathy in their recipients, both tumours were accompanied by marked thrombocytopenia and a gradual increase in blood fibrinogen level. These changes appeared during the period of lung metastatic growth after i.m. implantation of tumour cells (Poggi *et al.*, 1977; Chmielewska *et al.*, 1980) but it is not yet clearly established whether the metastases are indeed responsible for them.

In the 3LL system, thrombocytopenia, hyperfibrinogenaemia and haemolytic microangiopathic anaemia only occurred in experimental conditions where the primary tumour was present; they were not seen during lung colony growth after i.v. injection of cancer cells or during metastatic growth from i.m. implanted 3LL cells, if the primary had been surgically removed (Poggi *et al.*, 1980). Therefore, the pathogenesis of these changes remains open to investigation.

Recently, in several murine tumour models, sublines with different metastatic potential have been derived from the same parent line. This system may represent an interesting new tool with which to study biological processes associated with cancer-cell dissemination (Poste & Fidler, 1980). In this paper we have investigated the blood coagulation changes during the growth and metastasis of a murine fibro-

Correspondence to: Maria Benedetta Donati, M.D., Istituto di Ricerche Farmacologiche "Mario Negri", Via Eritrea 62, 20157 Milan, Italy.

TABLE.—*Metastasizing capacity of the mFS6 sarcoma and its sublines from metastases**

| Tumour line | MST† (days) | Day on which 50% of injected mice showed a palpable tumour | Mice with metastases/ total | No. metastases/ mouse (±s.e.) | Metastasis weight/ mouse (mg±s.e.) |
|---|---|---|---|---|---|
| mFS6 | 33 (25–50) | 14 | 17/32 | 3·3±0·3 | 18·2±5·4 |
| $M_4$ | 36 (30–49) | 13 | 13/14§ | 16·7±3·6‡ | 122·5±38·5‡ |
| $M_8$ | 35 (26–47) | 13 | 1/16§ | 1·0 | 0·5 |
| $M_9$ | 36 (25–52) | 15 | 0/15§ | — | — |

* $10^4$ tumour cells were injected i.m. and metastases were examined at death (Giavazzi *et al.*, 1980).
† Median survival time (with range).
‡ $P < 0·01$ compared to mFS6, Duncan's new multiple-range test.
§ $P < 0·01$ compared to primary mFS6, Fisher's exact test.

sarcoma and of 3 of its sublines with different metastatic capacity.

### MATERIALS AND METHODS

*Animals and tumours.*—Male C57BL/6J mice weighing 20–25 g at the start of the experiment were obtained from Charles River, Calco, Italy. The benzo(a)pyrene-induced mFS6 sarcoma, previously described in detail (Mantovani, 1978) spontaneously metastasizes to the lungs in about half of the i.m. injected syngeneic C57BL/6 hosts. It was used at its 10–20th passage. Cells from mFS6 and 3 sublines obtained from spontaneous lung nodules ($M_4$, $M_8$, $M_9$) were studied (Giavazzi *et al.*, 1980). The metastatic capacity of these sublines is shown in the Table. Tumours obtained 2–3 weeks after implantation were minced with scissors and disaggregated by exposure to 0·1% trypsin in Eagle's basal medium (BEM). The cells were washed twice with 50 ml BEM and resuspended in the same medium at the desired concentration. The tumour-cell suspension ($10^4$ cells) was injected i.m. in the right hind thigh of syngeneic mice. At spontaneous death, the number and weight of lung secondaries were measured as previously described (Mantovani, 1978).

*Blood coagulation assays.*—For each series of experiments, groups of 4 tumour-implanted mice from mFS6, $M_4$, $M_8$ and $M_9$ and from control animals, were killed at various intervals during the whole period of tumour development. In some experiments blood was collected by intracardiac puncture from open-chested animals under light ether anaesthesia. For anticoagulation, 9 parts of blood were mixed directly with 1 part 0·126M trisodium citrate in a disposable plastic syringe.

In other experiments native blood was collected from the retro-orbital plexus by means of a 20μl Konstriktions pipette (H. Pedersen, Oslo, Norway).

Blood platelets were counted by phase microscopy after dilution of blood with ammonium oxalate with a capillary standardized pipetting system (Unopette, Becton Dickinson Italia, Novate Milanese, Italy).

Blood fibrinogen concentration was measured by the Fibrin Polymerization Time (FPT) test adapted to mouse blood (Poggi *et al.*, 1977).

For activated partial thromboplastin time (APTT) and prothrombin time (PT) platelet-poor plasma (PPP) was obtained by centrifuging citrated blood in an Eppendorf centrifuge for 3 min at 12,000 *g*.

APTT was measured using Thrombofax (Ortho Cilag Chemie, Milan, Italy) as the platelet substitute and 0·5% Kaolin as activating material.

PT was measured using commercially available rabbit-brain thromboplastin (Hyland, Profarco, Milan, Italy (Poggi *et al.*, 1979).

### RESULTS

Fig. 1 shows the evolution of platelet counts and fibrinogen levels during the

FIG. 1.—Course of blood platelet count and blood fibrinogen level in mice at different intervals during growth of mFS6 or its sublines. Each result is expressed as a percentage of the value obtained from a paired control animal, tested simultaneously. Means ± s.e. of data from 4 animals per group.

development of mFS6 and its 3 sublines. In all instances the platelet count started to drop during the 3rd week after tumour-cell implantation, and gradually fell to 20–30% of the controls tested simultaneously. In animals implanted with the parent line or $M_8$ or $M_9$ sublines, blood fibrinogen was already higher than in controls one week after implantation, decreased one week later and increased again in the subsequent part of the observation period. In M4-bearing mice a progressive increase in blood fibrinogen level was observed, starting from the second week after tumour implantation.

Fig. 2 shows the PT and APTT values at 3 intervals during metastatic growth of mFS6 and its sublines. As regards PT, no changes were observed in any of the groups; the same was true for APTT, though with somewhat greater fluctuation, never exceeding 30–40% of control values.

FIG. 2.—PT and APTT ratios in plasma of mice at 3 intervals during metastatic growth of mFS6 or its sublines. Results are expressed as the ratio between clotting time in each tumour-bearing animal and its paired control. Means ± s.e. of data from 4 animals per group.

### DISCUSSION

This study shows that in a benzopyrene-induced fibrosarcoma of mice, the development of the tumour and the appearance of lung metastases was accompanied by a drop in blood platelet count and an increase in fibrinogen levels. In contrast, no significant changes were observed in PT and APTT, two tests which assess the extrinsic and intrinsic blood clotting pathways respectively. This indicates that the reduction in blood platelet count was not accompanied by clear signs of hypocoagulability, as would occur if the thrombocytopenia were due to consumption coagulopathy.

In the only two other murine metastasizing tumours investigated so far, the 3LL and the JW sarcoma, thrombocytopenia also occurred. For the 3LL, although low-grade intravascular coagulation was suspected, kinetic studies with radiolabelled platelets and marrow examinations clearly indicated that the drop in platelet count was due to impaired synthesis rather than increased consumption (Poggi *et al.*, 1977).

In the JW sarcoma, thrombocytopenia was not accompanied by any signs of intravascular coagulation (Chmielewska *et al.*, 1980). The increase in fibrinogen level observed in mFS6 appears to be due more to an acute-phase reaction than to a synthetic response in low-grade intravascular clotting. This contention is indirectly supported by the fact that hyperfibrinogenaemia has been reported in several rat and murine tumours, in association with either increased or normal fibrinogen turnover (Hilgard *et al.*, 1973; Poggi *et al.*, 1977; Chmielewska *et al.*, 1980) and with different patterns of fibrin deposition around the tumour. That thrombocytopenia and hyperfibrinogenaemia, at least in the 3LL model, were not due to consumption coagulopathy is further indicated by the fact that they were not corrected by anticoagulants or platelet aggregation inhibitors given chronically to tumour-bearing mice (Poggi *et al.*, 1980).

PT and APTT were normal also in the 3LL and JWS models. Thus mFS6, a chemically induced tumour, provoked essentially the same haemostatic changes as 3LL and JWS, of spontaneous origin.

The availability of cells derived from the same parent line but with different metastatic capacity, enables us to study metastasis-related biological properties with new perspectives. The sublines of mFS6 used in this study had markedly different metastatic potential (Giavazzi *et al.*, 1980) $M_8$ and $M_9$ being virtually unable to give metastases and $M_4$ being much more metastatic than the parent line. Our results indicate that, regardless of their dissemination capacity, the mFS6 sublines induced the same haemostatic changes in the host as the parent line. Since these cells did not differ as regards primary growth, our observations strongly support the concept that thrombocytopenia and hyperfibrinogenaemia are induced by the presence of the primary tumour rather than by metastatic nodules. It has indeed been reported in the 3LL system that thrombocytopenia and hyperfibrinogenaemia were only seen when the primary tumour was present, and were not modified by a treatment, such as warfarin anticoagulation, which selectively reduced metastatic growth (Poggi *et al.*, 1980).

When tested for their procoagulant activity *in vitro*, the $M_8$ and $M_9$ sublines showed a much greater clot-promoting capacity than $M_4$ or the parent line (Colucci *et al.*, 1980). Since such discrepancies were not reproduced when the same cells were given *in vivo* to mice, we conclude that no simple correlation exists between cancer-cell procoagulant activity as measured *in vitro* and the respective tumour-associated changes in the host's haemostatic system.

In conclusion, this study shows that some haemostatic changes may occur in animals bearing an experimental tumour whatever the pattern of its spontaneous metastasis.

This work was performed within the framework of the Cell-fibrin Interactions Subgroup of the Tumor Invasion Group of EORTC. The partial support of Italian National Research Council (Contract 80.01621.96), of NIH (Grant NIH PHRB-1R01 CA 12764-01 National Cancer Institute) and of Associazione Italiana per la Ricerca sul Cancro is gratefully acknowledged. Thrombin (Topostasine) was a gift from Dr Priore, Roche, Milano, Italy. Judith Baggott, Gigliola Brambilla, Paola Seminari, Vincenzo and Felice de Ceglie helped prepare this manuscript.

### REFERENCES

CHMIELEWSKA, J., POGGI, A., MUSSONI, L., DONATI, M. B. & GARATTINI, S. (1980) Blood-coagulation changes in JW Sarcoma, a new metastasizing tumor in mice. *Eur. J. Cancer* (in press).

COLUCCI, M., GIAVAZZI, R., ALESSANDRI, G., SEMERARO, N., MANTOVANI, A. & DONATI, M. B. (1980) Procoagulant activity of sarcoma sublines with different metastatic potential. *Blood* (in press).

DONATI, M. B. & POGGI, A. (1980) Malignancy and haemostasis. *Br. J. Haematol.*, **44**, 173.

GIAVAZZI, R., ALESSANDRI, G., SPREAFICO, F., GARATTINI, S. & MANTOVANI, A. (1980) Metastasizing capacity of tumour cells from spontaneous metastases of transplanted murine tumours. *Br. J. Cancer*, **42**, 462.

HILGARD, P., HOHAGE, R., SCHMITT, W, & KÖHLE. W. (1973) Microangiopathic haemolytic anaemia associated with hypercalcaemia in an experimental rat tumour. *Br. J. Haematol.*, **24**, 245.

MANTOVANI, A. (1978) Effects on *in vitro* tumor growth of muiine macrophages isolated from sarcoma lines differing in immunogenicity and metastasizing capacity. *Int. J. Cancer*, **22**, 741.

POGGI, A., DONATI, M. B. & GARATTINI, S. (1980) Fibrin and experimental cancer cell dissemination: Problems in the evaluation of experimental models. In *Malignancy and the Hemostatic System*. Eds Donati *et al.* New York: Raven Press. (In press.)

POGGI, A., KORNBLIHTT, L., DELAINI, F. & 4 others (1979) Delayed hypercoagulability after a single dose of adriamycin to normal rats. *Thromb. Res.*, **16**, 639.

POGGI, A., POLENTARUTTI, N., DONATI, M. B., DE GAETANO, G. & GARATTINI, S. (1977) Blood coagulation changes in mice bearing Lewis lung carcinoma, a metastasizing tumor. *Cancer Res.*, **37**, 272.

POSTE, G. & FIDLER, I. J. (1980) The pathogenesis of cancer metastasis. *Nature*, **283**, 139.

RASCHE, H. & DIETRICH, M. (1977) Hemostatic abnormalities associated with malignant diseases. *Eur. J. Cancer*, **13**, 1053.